# MIIP Inhibits Colorectal Cancer Progression by Modulating Neutrophils Infiltration and Neutrophil Extracellular Trap Formation

**DOI:** 10.1002/advs.78063

**Published:** 2026-09-29

**Authors:** Jiaxin Li, Jia Deng, Lin Sun, Shihui Wang, Huizhi Li, Jing Chen, Lipan Zhao, Xilin Shen, Yan Sun

**Affiliations:** ^1^ Department of Pathology National Clinical Research Center for Cancer Tianjin's Clinical Research Center for Cancer Tianjin Key Laboratory of Digestive Cancer Tianjin Medical University Cancer Institute & Hospital Tianjin China

**Keywords:** colorectal cancer, migration and invasion inhibitory protein, neutrophil extracellular traps, tumor progression, tumor‐associated neutrophils

## Abstract

Neutrophil extracellular traps (NETs) contribute to colorectal cancer (CRC) progression, but the underlying regulatory mechanism remains unknown. This study explored how migration and invasion inhibitory protein (MIIP) influences CRC progression by regulating tumor‐associated neutrophils (TANs) infiltration and NETs formation. On the basis of bioinformatics analysis and clinical CRC samples, we observed that a high density of neutrophils and a high abundance of NETs were correlated with low MIIP expression in CRC tissue as well as distant metastasis and poor prognosis. In vitro co‐culture assays revealed that conditioned medium from CRC cells with downregulated MIIP expression recruited neutrophils to form NETs and that NETs further enhanced the migratory and invasive abilities of CRC cells. Mechanistically, the results of this study demonstrated that MIIP directly bound to Protein Kinase R, which inhibited the Nuclear Factor kappa‐B pathway and Interleukin ‐ 8 expression and accordingly suppressed TANs infiltration and NETs formation. In addition, preliminary results from an intrasplenic mouse model of CRC liver metastasis suggested the potential therapeutic significance of Deoxyribonuclease I (DNase I, a NET inhibitor), and MIIP augmented the inhibition of CRC metastasis by DNase I. This study revealed that MIIP is a regulator of CRC cell‐neutrophil crosstalk and indicated that NETs are potential therapeutic targets in CRC.

## Graphical abstract

This diagram illustrates that MIIP suppresses tumor‐associated neutrophils infiltration and NETs formation by binding to PKR, which impairs the PKR–IKKβ interaction and attenuates NF‐κB/IL‐8 axis activation, thereby inhibiting colorectal cancer progression. This mechanism highlights a promising therapeutic target for CRC patients.

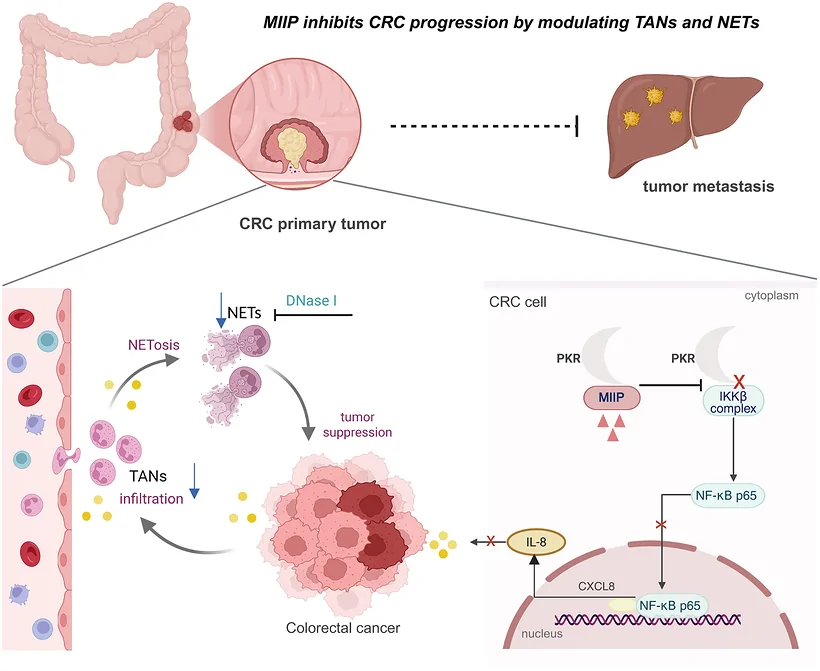

## 1 Introduction

Colorectal cancer (CRC) ranks third in incidence and second in mortality among all malignancies worldwide, and most deaths are ultimately attributable to liver metastasis [1, 2]. Tumor progression is driven not only by tumor cells but also by interplay with the tumor immune microenvironment (TIME) [3]. Although breakthrough results in solid tumors have been achieved using immune checkpoint inhibitors (ICIs), they are ineffective in approximately 85% of CRC patients with microsatellite stable (MSS)/proficient mismatch repair (pMMR) status, and most of these patients remain refractory [4, 5]. Therefore, identifying novel targets for improving survival rates of CRC patients is essential. The migration and invasion inhibitory protein (MIIP) has been demonstrated to inhibit tumor cell proliferation, migration, and invasion, thereby suppressing the progression of various tumors including glioma [6, 7], endometrial cancer [8], renal cancer [9], colorectal cancer [10, 11, 12] and breast cancer [13]. Our previous study revealed that MIIP haploinsufficiency induced chromosomal instability (CIN) and promoted CRC progression through disruption of the APC/C^Cdc20^‐mediated degradation of securin and cyclin B1 [11]. Recently, MIIP downregulation was shown to accelerate CRC progression by inducing adipocyte browning and the release of free fatty acids into the tumor microenvironment (TME) through the promotion of N‐glycosylation and AZGP1 oversecretion in CRC cells [14]. Moreover, MIIP downregulation increases IL10 secretion via the dsDNA–STING–NFκB2–IL10 signaling axis in CRC cells and drives M2 macrophage polarization in the CRC TME [12]. The results of these investigations collectively indicate that MIIP can not only inhibit the malignant behavior of tumor cells, but also perform multifaceted tumor‐suppressive functions by modulating the interaction between tumor cells and TME components.

Neutrophils are the most abundant leukocytes in human peripheral blood and constitute the first line of defense of the innate immune system against invading pathogens [15]. Tumor‐associated neutrophils (TANs) can exert antitumor effects (N1 phenotype) through direct cytotoxicity or indirect activation of adaptive immune responses [16, 17] or by exerting protumor effects (N2 phenotype) through the promotion of tumor cell proliferation, angiogenesis, ECM remodeling, distant metastasis, immune suppression, and neutrophil extracellular traps (NETs) formation [18, 19, 20, 21, 22, 23]. NETs are web‐like structures released by activated neutrophils, primarily composed of double‐stranded DNA (dsDNA), histones, and antimicrobial proteins such as myeloperoxidase (MPO) [24]. NETs can act as accomplices of neutrophils and play a primary role in promoting tumor progression through the entrapment of circulating tumor cells, remodeling of the extracellular matrix, and creation of a premetastatic niche [25, 26, 27, 28]. However, the mechanism underlying the modulation of TANs polarization and NETs formation remains unclear. Given their protumor role, targeting NETs has emerged as a promising therapeutic strategy. Deoxyribonuclease I (DNase I) degrades NETs by disrupting their DNA backbone [29] and was demonstrated to inhibit lung metastasis in murine models of melanoma and breast cancer [30, 31], suggesting that DNase I is a potential treatment method for cancer therapy.

In this study, we utilized an integrative approach combining bioinformatics analysis, clinical CRC specimens, an in vitro co‐culture system, and an intrasplenic mouse model of CRC liver metastasis to demonstrate the regulatory effect of MIIP in CRC cells on neutrophil infiltration and NETs formation, which expands the function of MIIP as a tumor suppressor through the modulation of TANs and NETs in the TIME. Mechanistically, we demonstrated that MIIP bound Protein Kinase R (PKR), which inhibited Nuclear Factor kappa‐B (NF‐κB) pathway activation and interleukin‐8 (IL‐8) expression, thereby suppressing neutrophil infiltration and reprogramming as well as NETs formation. In addition, the present findings suggest that DNase I is a potential treatment for inhibiting CRC liver metastasis and that MIIP might serve as a marker for DNase I treatment.

## 2 Results

### 2.1 MIIP Expression is Negatively Correlated With TANs Infiltration and NETs Formation in CRC Patients

To explore the correlation between neutrophil infiltration and MIIP expression in CRC, we performed a CIBERSORT algorithm‐based analysis using The Cancer Genome Atlas (TCGA) CRC database. The results revealed that *MIIP* expression was negatively correlated with the enrichment of neutrophils (Figure S1A,B). Then, using immunohistochemical staining (IHC) in 400 clinical CRC tissue samples, we demonstrated that the number of TANs was significantly greater in CRC tissue with low MIIP expression than in that with high MIIP expression (Figure 1A,B). In addition, the high density of TANs in CRC tissue was associated with distant tumor metastasis (Figure 1C; Table S1), poor progression‐free survival (PFS), and poor overall survival (OS) in CRC patients (Figure 1D).

**FIGURE 1:**
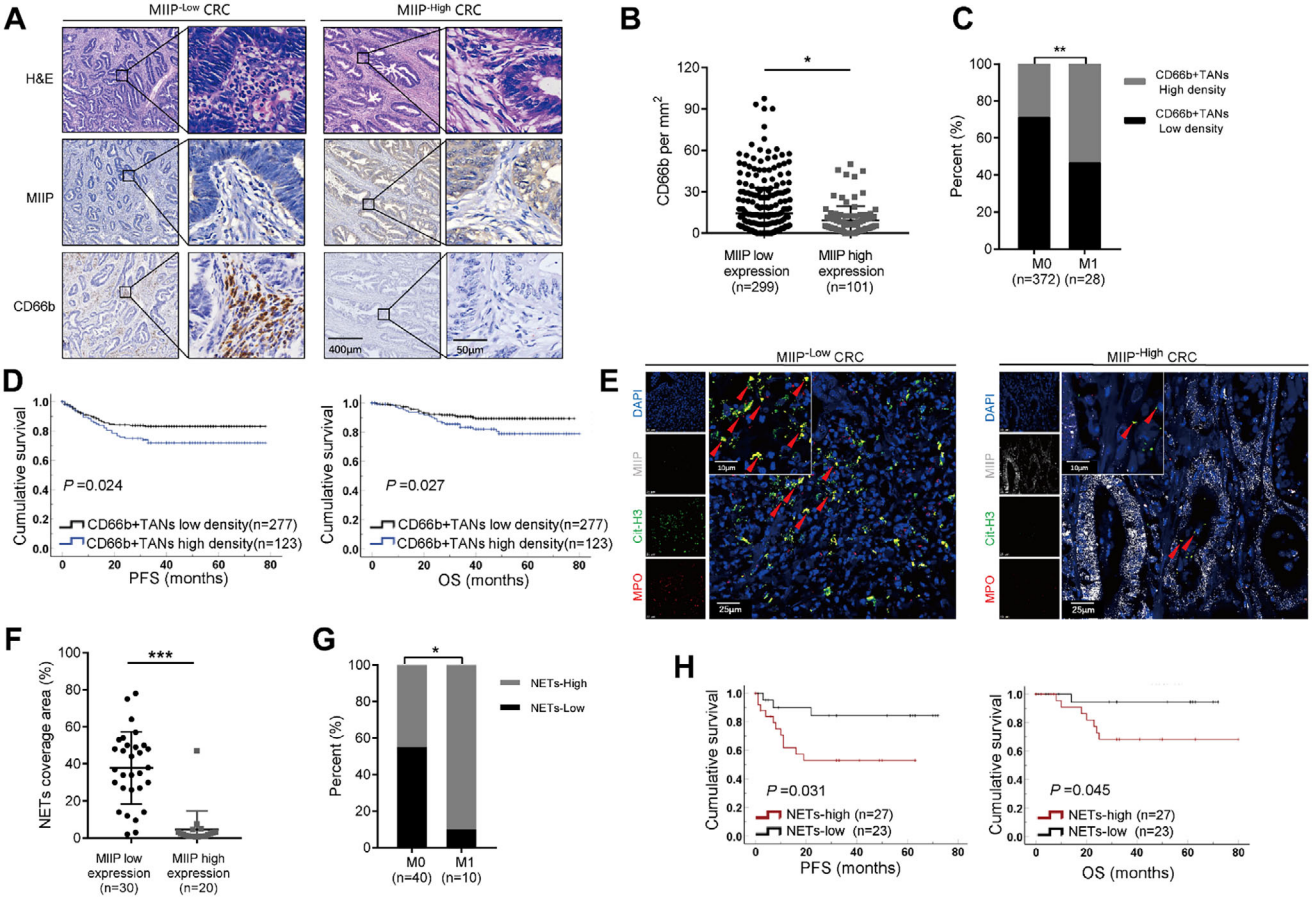
Low expression of MIIP is associated with increased TANs infiltration and NETs formation in CRC tissues from the Tianjin cohort. (A) Representative images of H&E and IHC staining for MIIP and CD66b^+^ TANs in clinical CRC samples (*n* = 400). Scale bars: 400 µm, 50 µm. (B) Comparison of intratumoral CD66b^+^ TANs between the groups with high and low MIIP expression (*n* = 400). (C) The relationship between CD66b^+^ TANs and CRC distant metastasis (*n* = 400). (D) Survival analyses grouped by CD66b^+^ TANs infiltration (*n* = 400). (E) Representative IF images for MPO (red), Cit‐H3 (green), MIIP (gray), and DAPI (blue) in tissue from CRC patients. Red arrowheads indicate NETs (*n* = 50). Scale bars: 25, 10 µm. (F) Comparison of the NETs density between the high‐ and low‐MIIP groups. (G) The relationship between NETs and CRC distant metastasis (*n* = 50). (H) Survival analyses grouped by NETs density (*n* = 50). ^*^, *p* < 0.05; ^**^, *p* < 0.01; ^***^, *p* < 0.001. Statistical significance was assessed using Student's t‐test in (B); the chi‐square test in (C); the Mann‐Whitney U test in (F); Fisher's exact test in (G); and the log‐rank test in (D, H).

Given that NETs serve as an indispensable functional modality through which neutrophils affect the TME, we further examined the correlation between NETs‐related gene signatures [32, 33] and MIIP expression across the TCGA CRC and pan‐cancer datasets. MIIP expression was negatively associated with NETs‐related signatures (Figure S1C). Immunofluorescence (IF) staining for Cit‐H3 and MPO, which serve as well‐established biomarkers for NETs formation [30, 34], revealed that NETs abundance was markedly higher in CRC tissue with low MIIP expression than in that with high MIIP expression (Figure 1E,F). Furthermore, we found that a high density of NETs formation was correlated with distant metastasis (Figure 1G; Table S2) as well as poor PFS and poor OS in CRC patients (Figure 1H).

### 2.2 MIIP Inhibits Neutrophil Migration, N2 Polarization, and NETs Formation In Vitro

By examining the endogenous expression of MIIP in 17 CRC cell lines (Figure S2A), we selected SW620 cells with low MIIP expression for overexpression experiments and HCT116 cells with high MIIP expression for knockdown assays. Considering that SW620 cells were originally obtained from a metastatic tumor in the same patient from whom SW480 cells were obtained, we also chose SW480 cells with relatively high MIIP expression for knockdown studies. We validated the effects of MIIP overexpression and knockdown in the three stably transfected CRC cell lines (Figure S2B,C). In addition, we isolated fresh neutrophils from the peripheral blood (PB) of healthy adults and verified that the purity exceeded 90% (Figure S2D). We performed a series of experiments using a co‐culture of the above CRC cells or their conditioned medium (CM) and neutrophils (Figure 2A). Flow cytometry revealed no differences in apoptosis when neutrophils were treated with CM from CRC cells with different MIIP expressions (Figure S2E). In a neutrophil chemotaxis assay, we found that MIIP overexpression in CRC cells significantly decreased the migration of neutrophils toward the CRC CM, whereas knockdown of MIIP in CRC cells significantly increased the migration of neutrophils toward the CRC CM (Figure 2B). IF staining for NETs formation revealed that CM from MIIP‐knockdown CRC cells was more potent, while CM from MIIP‐overexpressing cells was less potent at inducing NETs formation (Figure 2C,D). MPO‐DNA levels, detected by Enzyme‐Linked Immunosorbent Assay (ELISA) and used as a biomarker of NETs [34, 35], were also significantly increased in neutrophils treated with CRC CM (Figure 2E). Moreover, the SYTOX Green assay, another method to detect dsDNA [36], revealed consistent results (Figure S2F) with the MPO‐DNA ELISA assay.

**FIGURE 2:**
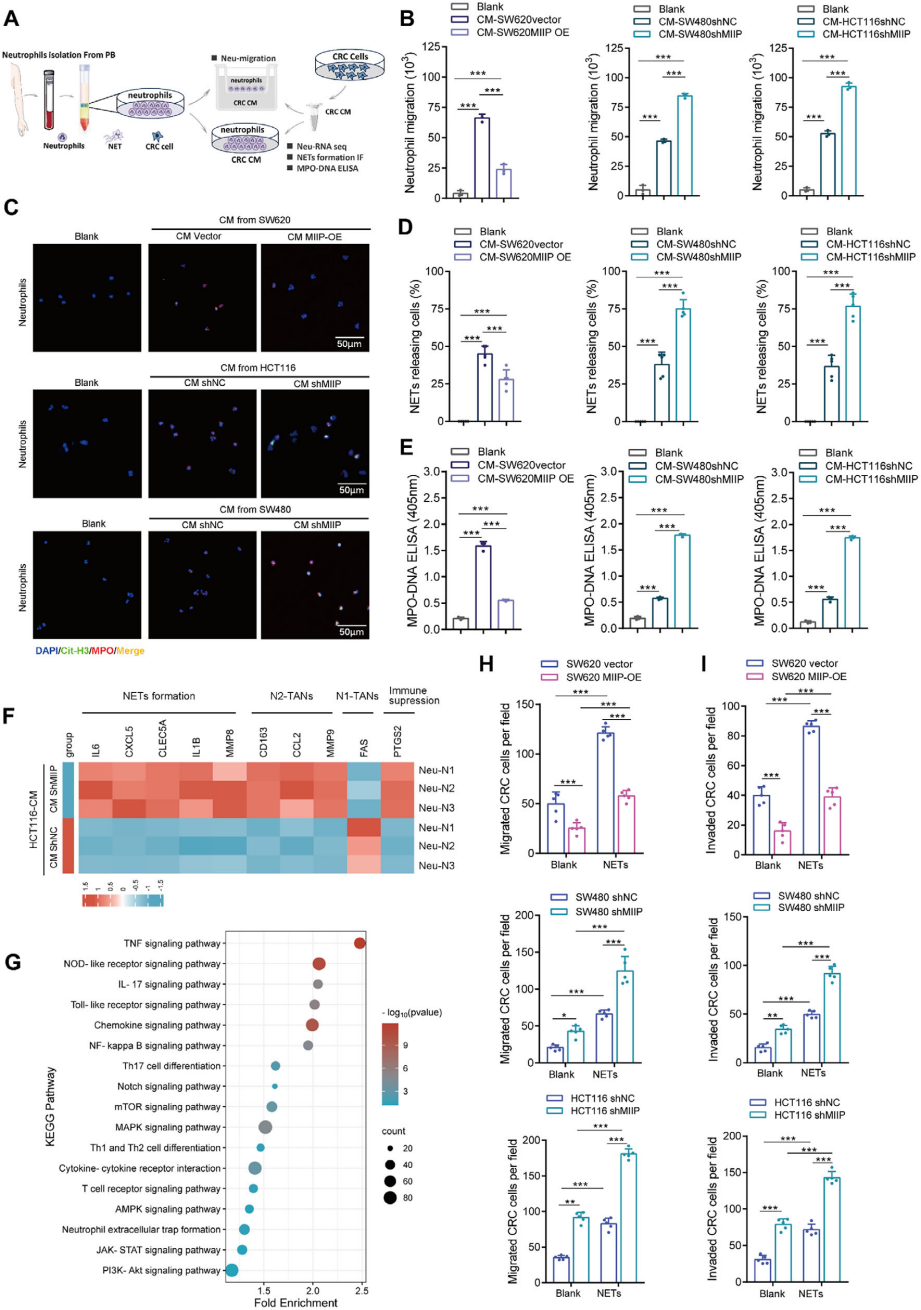
MIIP in CRC cells restrains neutrophil infiltration and NETs formation in vitro. (A) Diagram of in vitro co‐culturing experiments for neutrophils isolated from the peripheral blood of healthy adults and CM from CRC cells. (B) The number of migrated neutrophils was evaluated via a neutrophil chemotaxis assay (*n* = 3). (C, D) Representative images and statistical quantification of IF staining for MPO (red), Cit‐H3 (green), and DAPI (blue) to show NETs formation (*n* = 5). Scale bar, 50 µm. (E) ELISA analysis of MPO‐DNA for NETs formation (*n* = 3). (F) Heatmap of RNA‐seq data for neutrophils cocultured with CRC CM (*n* = 3 per group). (G) KEGG pathway enrichment of the differentially expressed genes (DEGs) based on RNA‐seq data of neutrophils. (H, I) Quantification of the results of the transwell migration and invasion assays of CRC cells cocultured with or without NETs (*n* = 5). All the data are shown as the mean ± SD; ^*^, *p* < 0.05; ^**^, *p* < 0.01; ^***^, *p* < 0.001. Statistical significance was assessed using one‐way ANOVA in (B, D, and E) and two‐way ANOVA in (H, I).

RNA sequencing (RNA‐seq) of neutrophils cocultured with CM from HCT116 shNC or HCT116 shMIIP cells revealed distinct transcriptome profiles between the two groups of neutrophils (Figure S2G). N1‐associated genes (such as *Fas*) were significantly downregulated, and N2 neutrophil phenotype‐associated genes [37, 38, 39], including *CD163*, *CCL2* and *MMP9*, were upregulated in Neu‐HCT116 shMIIP CM compared with those in Neu‐HCT116 shNC CM (Figure 2F). We also found that the expression of several NETs formation‐associated genes [40, 41, 42], including *IL‐6*, *CXCL5*, *CLEC5A*, *IL‐1B* and *MMP8*, was significantly upregulated in Neu‐HCT116 shMIIP cells (Figure 2F). Further Kyoto Encyclopedia of Genes and Genomes (KEGG) pathway analysis demonstrated that canonical signaling pathways involved in NETs formation [43, 44], such as the NOD‐like receptor signaling pathway and Toll‐like receptor signaling pathway, were enriched (Figure 2G). Collectively, these findings indicate that downregulation of MIIP expression in CRC cells enhances neutrophil chemotaxis, reprograms neutrophils into a protumoral phenotype, and promotes NETs formation.

We subsequently evaluated the effects of neutrophil‐derived NETs on CRC cells in a coculture system. MIIP overexpression inhibited CRC cell migration and invasion, but this suppression was reversed upon coculture with NETs; conversely, MIIP knockdown promoted migration and invasion of CRC cells, which were further enhanced by NETs (Figure 2H,I; Figure S2H,I). CCK‐8 proliferation assays demonstrated that NETs promoted CRC cell proliferation, particularly in cells expressing MIIP at low levels (Figure S2J).

### 2.3 MIIP Suppresses Neutrophil Migration and NETs Formation by Decreasing IL‐8 Expression in CRC Cells

To explore the key cytokines in CRC CM, we performed a human cytokine array analysis to compare the levels of secreted proteins in the CM of SW620 cells with different levels of MIIP expression. Among the cytokines evaluated, IL‐8 (encoded by *CXCL8*) was the most downregulated when MIIP was overexpressed (Figure 3A). ELISA confirmed that compared with control cells, MIIP‐overexpressing SW620 cells secreted less IL‐8, whereas MIIP‐knockdown SW480 and HCT116 cells secreted more IL‐8 (Figure 3B). RNA sequencing also revealed *CXCL8* as one of the genes whose expression was significantly upregulated in MIIP‐knockdown HCT116 cells (Figure 3C), which was verified by RT–qPCR in three cell lines (Figure 3D). A rescue functional assay further demonstrated that the recombinant IL‐8 protein effectively reversed the inhibitory effect of the CM from MIIP‐overexpressing CRC cells on neutrophil migration and NETs formation, whereas the addition of an anti‐IL‐8‐neutralizing antibody to CM from CRC cells with low MIIP expression partially inhibited neutrophil migration and NETs formation (Figure 3E–H). Taken together, these results suggest that IL‐8 is a key mediator of MIIP‐ regulated neutrophil migration and NETs formation in CRC.

**FIGURE 3:**
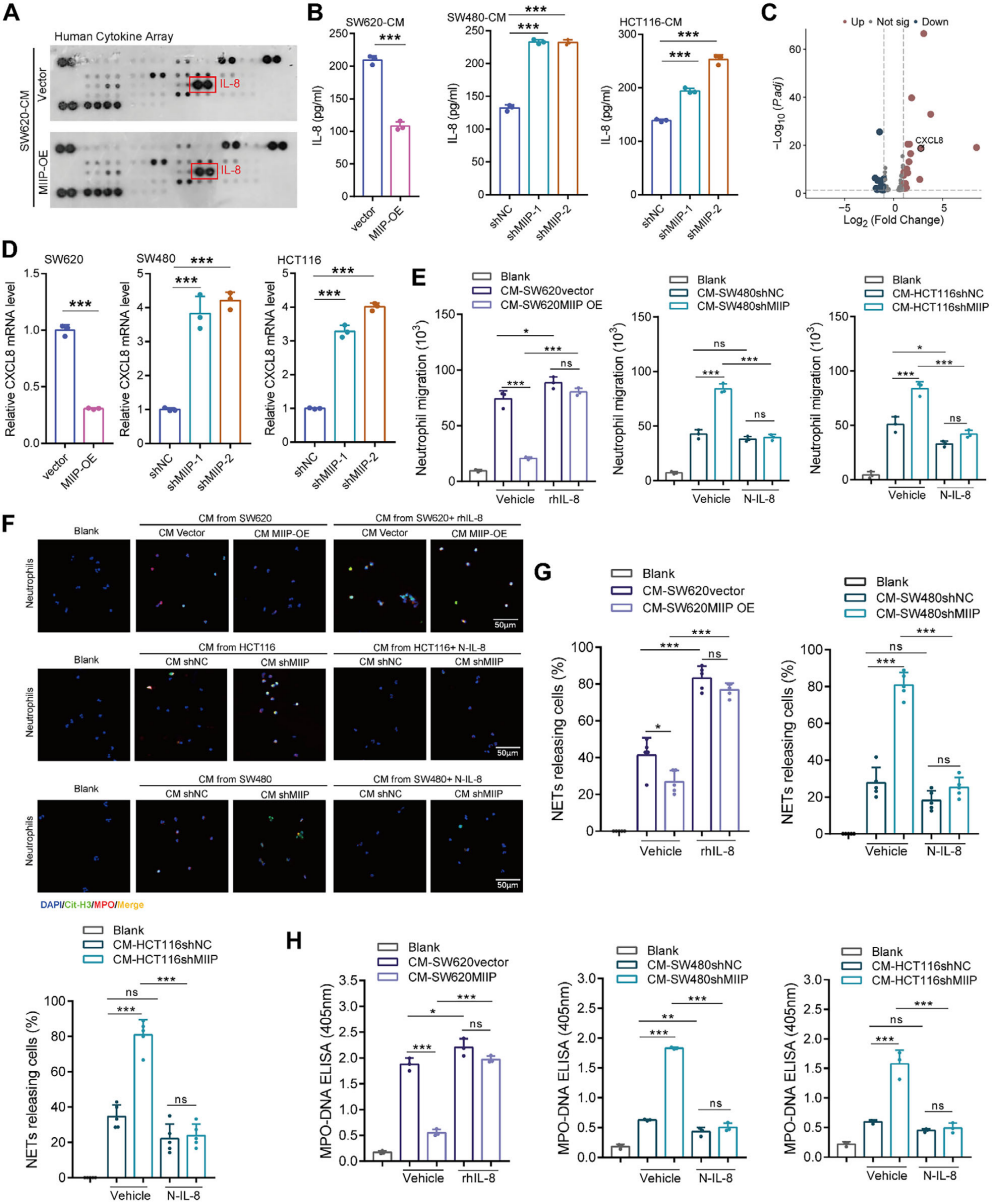
MIIP in CRC cells inhibits neutrophil infiltration and NETs formation through IL‐8. (A) Human cytokine array of the CM collected from SW620 cells with vector control and MIIP overexpression. (B) ELISA quantification of secreted IL‐8 protein levels in CM derived from three CRC cell lines with differential MIIP expression (*n* = 3). (C) Volcano plot of DEGs from RNA‐seq analysis of CRC cell lines with varying levels of MIIP expression (*n* = 3 per group). (D) *CXCL8* mRNA expression in human CRC cells with different levels of MIIP expression was tested by RT–qPCR (*n* = 3). (E) Quantification of neutrophils that migrated to CRC CM with or without recombinant human IL‐8 protein (rhIL‐8). Quantification of neutrophils that migrated to the CRC CM with or without anti‐IL‐8 neutralizing antibody (N‐IL‐8) (*n* = 3). (F–H) Representative images and statistical quantification of IF staining (*n* = 5) and MPO‐DNA ELISA (*n* = 3) for NETs formation after neutrophils were co‐cultured with the CM from SW620 cells treated with recombinant IL‐8 protein and with the CM from SW480 and HCT116 cells treated with an anti‐IL‐8 neutralizing antibody. Scale bar, 50 µm. All the data are shown as the mean ± SD; ^*^, *p* < 0.05; ^**^, *p* < 0.01; ^***^, *p* < 0.001. NS, not significant. Statistical significance was assessed using Student's t‐test and two‐way ANOVA in (B, D, E, G, and H).

### 2.4 MIIP Regulates IL‐8 Through the NF‐κB p65 Signaling Pathway in CRC Cells

To explore how MIIP modulates IL‐8 expression, we performed KEGG analyses and gene set enrichment analysis (GSEA) on RNA‐seq data from HCT116 cells with different MIIP expression. The results indicated that the NF‐κB signaling pathway was the most significantly enriched pathway in the group with low MIIP expression levels (Figure 4A,B). Furthermore, Western blotting revealed that the phosphorylation of NF‐κB p65 was significantly decreased in MIIP‐overexpressing cells but increased in MIIP‐knockdown cells (Figure 4C; Figure S3A). Consistent results were obtained in mouse CRC cell lines (Figure S3B–D). In the orthotopic CRC mouse model, MIIP overexpression significantly reduced the expression levels of p‐p65 and IL‐8 in tumor cells and the infiltration of Ly6G^+^ neutrophils (Figure 4D,E), as well as NETs formation (Figure 4F). Treatment with an NF‐κB inhibitor reversed the increased effect of MIIP downregulation on the phosphorylation of NF‐κB p65 and IL‐8 (Figure 4G–I). Moreover, CM collected from CRC cells treated with an NF‐κB inhibitor reversed the effects of low MIIP expression on neutrophil chemotaxis (Figure 4J) and NETs formation (Figure 4K–M). Collectively, the function of MIIP as a tumor suppressor in CRC is partly due to MIIP‐mediated inhibition of neutrophil infiltration and NETs formation through the NF‐κB p65/IL‐8 signaling pathway.

**FIGURE 4:**
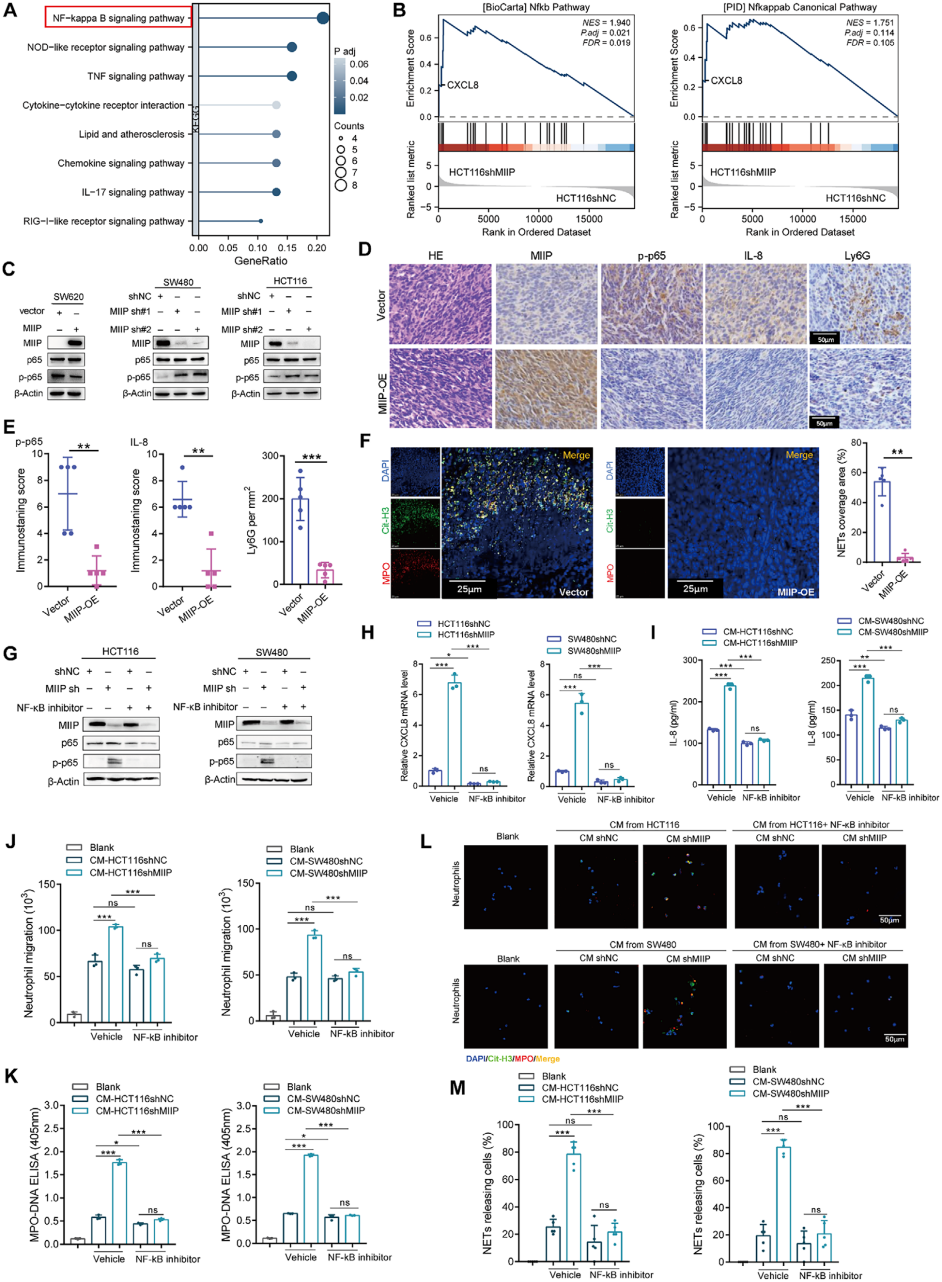
MIIP in CRC cells decreases neutrophil infiltration and NETs formation through NF‐κB p65/IL‐8 signaling. (A) Bubble plot of the enriched KEGG pathways showing that the NF‐κB signaling pathway was enriched in HCT116 cells (marked with a red border). (B) GSEA revealed that MIIP knockdown in HCT116 cells significantly activated the NF‐κB signaling pathway. (C) Western blotting analysis of key proteins involved in the NF‐κB p65 signaling pathway in human CRC cells with different levels of MIIP expression. (D) Representative images of H&E and IHC staining for MIIP, p‐p65, IL‐8, and Ly6G in tumors from the cecum of an orthotopic CRC mouse model between the MIIP‐overexpressing group and the control group (n = 5 per group). Scale bar, 50 µm. (E) Statistical analysis of Ly6G^+^ neutrophils and the expression of p‐p65 and IL‐8 in different groups. (F) Representative images and statistical quantification of IF staining for MPO (red), Cit‐H3 (green), and DAPI (blue) in tumor tissue from the cecum and comparisons between the two groups (*n* = 5). Scale bar, 25 µm. (G) Western blotting of NF‐κB p65 and NF‐κB p‐p65 in CRC cells treated with or not treated with an NF‐κB inhibitor (Bay 11–7082, 10 µM). (H) RT‐qPCR of *CXCL8* mRNA in CRC cells treated or not treated with an NF‐κB inhibitor (*n* = 3). (I) ELISA of IL‐8 in CM from CRC cells treated or not treated with an NF‐κB inhibitor (*n* = 3). (J) Quantification of neutrophils that migrated to the CM from CRC cells treated or not treated with an NF‐κB inhibitor (*n* = 3). (K–M) ELISA of MPO‐DNA and representative images and statistical quantification of IF staining for NETs formation after neutrophils were cultured with CM from SW480 and HCT116 cells treated or not treated with an NF‐κB inhibitor (*n* = 3–5). Scale bar, 50 µm. All the data are shown as the mean ± SD; ^*^, *p* < 0.05; ^**^, *p* < 0.01; ^***^, *p* < 0.001. NS, not significant. Statistical significance was assessed using Student's t‐test and the Mann‐Whitney U test in (E); the Mann‐Whitney U test in (F); and two‐way ANOVA in (H, I, J, K, and M).

### 2.5 MIIP Suppresses NF‐κB p65/ IL‐8 Signaling Through Interaction With PKR

To further explore the mechanism through which MIIP regulates the NF‐κB p65‐mediated IL‐8 signaling pathway, we performed an immunoprecipitation‐mass spectrometry (IP–MS) assay (Figure 5A). We identified 136 proteins that specifically interact with MIIP in the MIIP‐OE group through IP–MS. Among these top 10 proteins ranked by mass spectrometry metrics, three proteins were related to the regulation of NF‐κB signaling: STOML2, ZC3H11A, and PKR (Figure S4A). Molecular docking revealed that MIIP binds to the N‐terminal domain (residues 1–265) of PKR (Figure 5B; Figure S4B), which overlaps with the IKKβ‐binding domain required for NF‐κB pathway activation. Therefore, PKR was selected as our primary candidate protein. We subsequently confirmed the interaction between MIIP and PKR through endogenous coimmunoprecipitation (Co‐IP) experiments in HCT116 cells (Figure 5C) and forward and reverse exogenous Co‐IP experiments in 293T cells (Figure 5D). Through Co‐IP assays using PKR and MIIP truncated plasmids designed on the basis of the molecular docking prediction results (Figure S4B), we demonstrated that the primary binding site is mainly located within the 1–265 domain of PKR and within amino acid residues 313–388 of MIIP (Figure 5E,F). Moreover, MIIP not only inhibited PKR protein expression (Figure 5G), but also attenuated the interaction between PKR and its downstream effector IKKβ (Figure 5H), thereby impairing NF‐κB p65 signaling activation.

**FIGURE 5:**
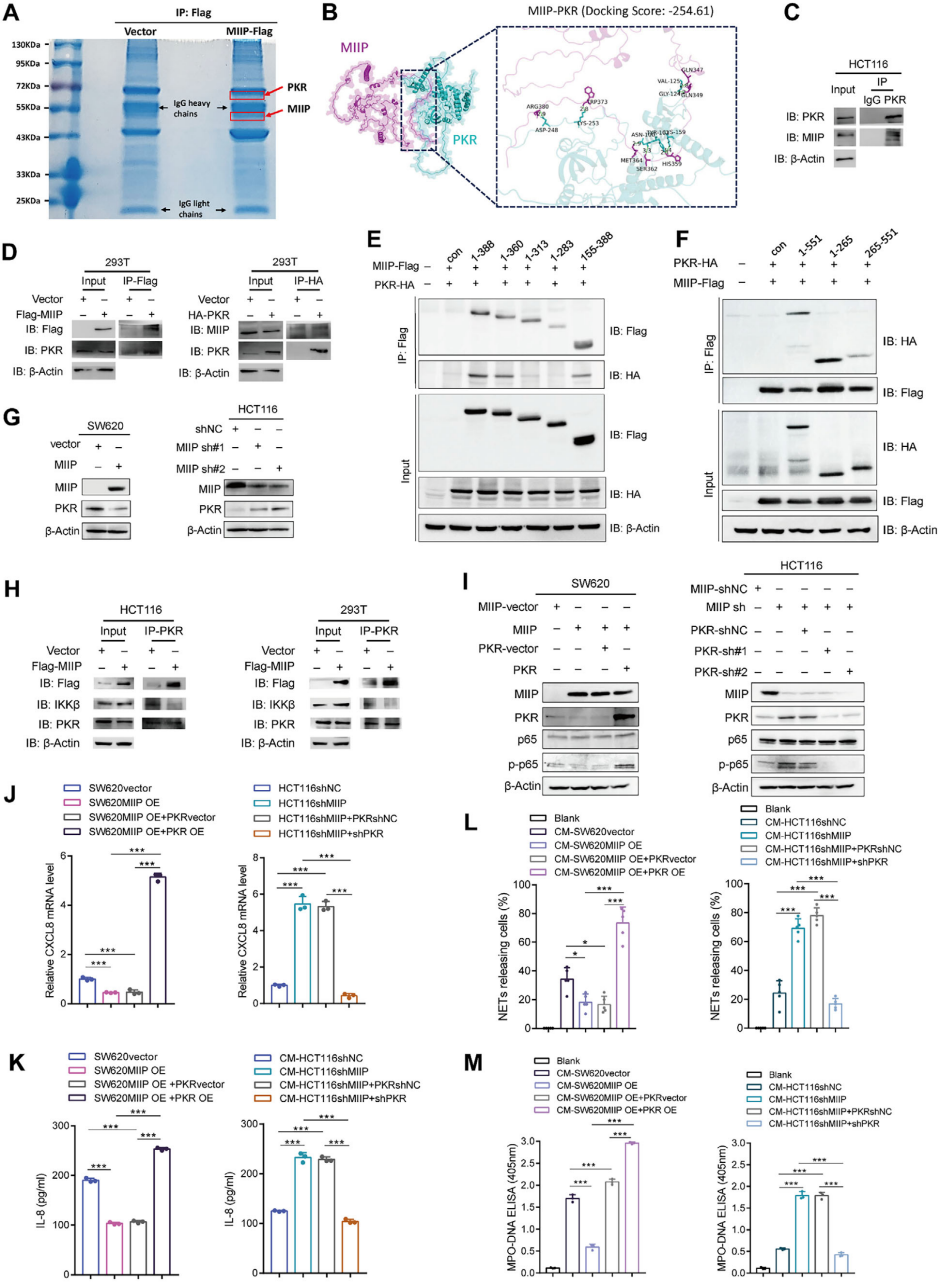
MIIP modulates the NF‐κB p65/IL‐8 signaling pathway ‐mediated neutrophil infiltration and NETs formation through interactions with PKR. (A) IP–MS revealed PKR as a MIIP‐interacting protein. (B) The binding structure of MIIP and PKR was predicted using HDOCK molecular docking software. (C) Endogenous interaction between MIIP and PKR were detected via Co‐IP in HCT116 cells. (D) 293T cells were cotransfected with MIIP‐Flag and PKR‐HA, and reverse and forward Co‐IP analyses were performed. (E) Co‐IP assays were used to determine the PKR‐binding region of MIIP. (F) Co‐IP assays revealed that the PKR binding site for MIIP encompasses its IKKβ‐binding domain. (G) The expression of PKR was determined by Western blotting in CRC cells with different levels of MIIP expression. (H) Co‐IP was performed to examine the effect of MIIP expression on the interaction between PKR and IKKβ. (I–M) Double‐stable transgenic CRC cell lines were used in a rescue assay to demonstrate that the regulatory effects of MIIP on the NF‐κB p65‐mediated IL‐8 signaling pathway (I–K) and NETs formation (L, M) were dependent on PKR (*n* = 3–5). All the data are shown as the mean ± SD; ^*^, *p* < 0.05; ^**^, *p* < 0.01; ^***^, *p* < 0.001. NS, not significant. Statistical significance was assessed using two‐way ANOVA in (J–M).

To further validate the effect of MIIP on NF‐κB p65 signaling through PKR, we performed rescue experiments utilizing double‐stable transgenic CRC cell lines. PKR overexpression reversed the inhibitory effect of MIIP overexpression on NF‐κB activation and IL‐8 secretion, and PKR knockdown attenuated the increase in NF‐κB activation and IL‐8 secretion in MIIP knockdown cells (Figure 5I–K). Moreover, the effects of MIIP on NETs formation were reversed by PKR overexpression (Figure 5L,M; Figure S4C). The above results revealed that MIIP bound to the N‐terminal domain (aa 1–265) of PKR via its C‐terminal region (aa 313–388), thereby inhibiting the activation of the NF‐κB p65/IL‐8 signaling pathway and suppressing NETs formation.

### 2.6 MIIP Enhances the Inhibitory Effect of DNase I on CRC Metastasis In Vitro and In Vivo

We further explored the potential therapeutic significance of DNase I (a NET inhibitor). In vitro assays revealed that the presence of NETs in the co‐culture system significantly enhanced the migration and invasion of CRC cells, particularly those with low MIIP expression, but these effects were abolished by DNase I treatment (Figure S5A,B; Figure 6A,B). We further generated a murine liver metastasis model by splenic injection of mouse CT26 cells with different levels of MIIP expression (Figure 6C). Compared with the control treatment, both the injection of MIIP‐overexpressing CRC cells and the administration of DNase I decreased the number of liver metastases (Figure 6D–G). Furthermore, the group injected with both MIIP‐overexpressing CRC cells and DNase I treatment had the fewest liver metastases. Multicolor IF staining of liver metastases also revealed that, compared with the control group (vehicle), the group treated with DNase I effectively cleared NETs from tumor tissues, and the number of NETs was the lowest in the group with both MIIP‐overexpressing CRC cells and those treated with DNase I (Figure 6H,I). In addition, compared with the control group, DNase I did not influence the body weight of the mice (Figure S5C) or lead to obvious histopathological abnormalities in mouse brains, hearts, kidneys, or lungs (Figure S5D), suggesting that DNase I has a favorable safety profile for CRC treatment in this mouse model.

**FIGURE 6:**
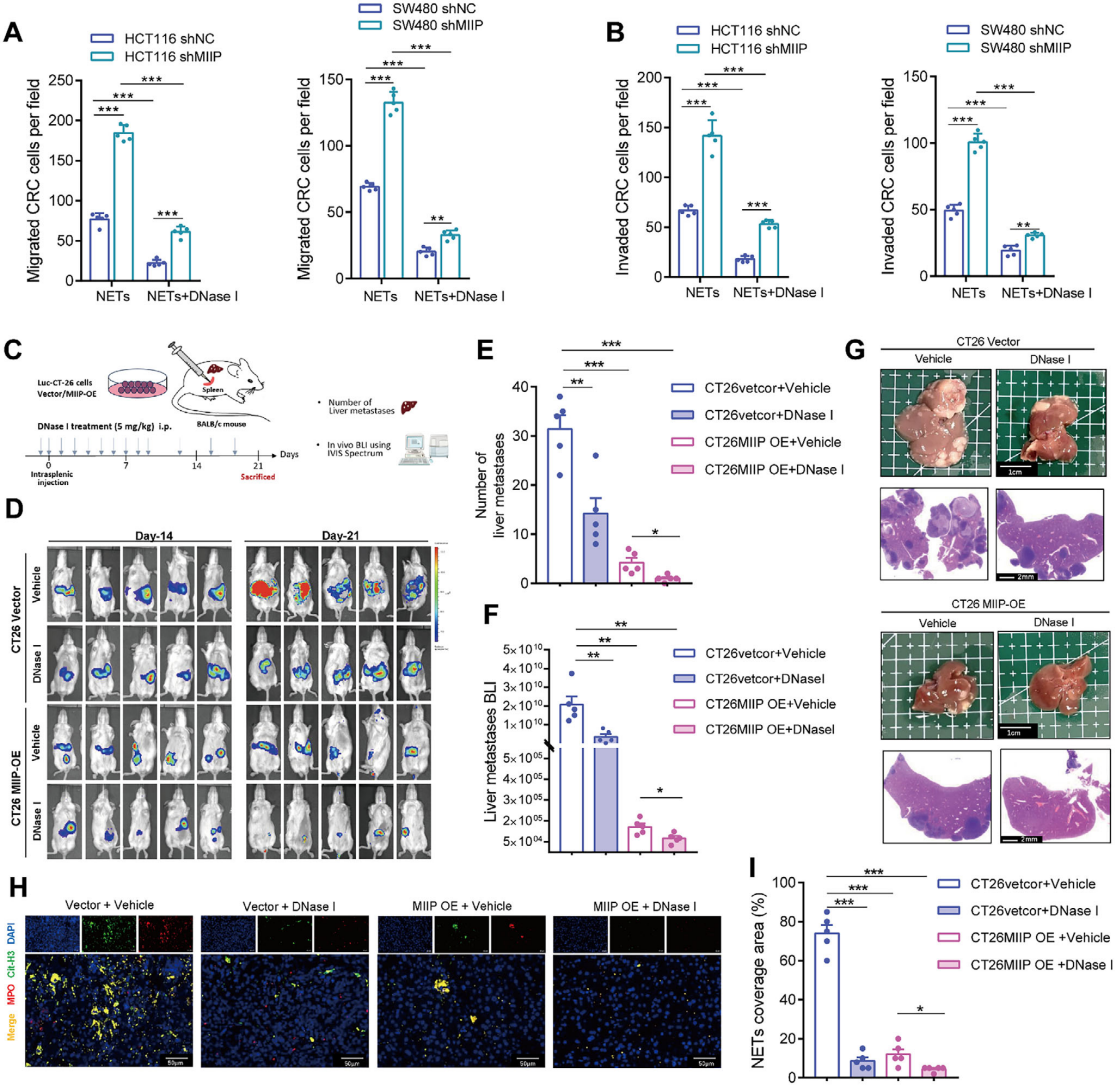
MIIP enhances the inhibition of CRC cell migration and invasion by DNase I (a NET inhibitor) in vitro and liver metastasis in vivo. (A) Quantification of the results of the Transwell migration assay of CRC cells after co‐culture with NETs and NETs + DNase I (*n* = 5). (B) Quantification of the results of the transwell invasion assay of CRC cells after co‐culture with NETs and NETs + DNase I (*n* = 5). (C) Schematic diagram of a murine splenic liver metastasis model established by injecting CT26 cells into the spleens of mice and mice treated with DNase I. (D) Bioluminescence imaging (BLI) of BALB/C mice injected with CT26 cells with different levels of MIIP expression in the spleen and treated or not treated with DNase I (*n* = 5 in each group; DNase I, 5 mg/kg). (E) Quantification of the BLI results and statistical analysis (*n* = 5). (F) Comparison of the numbers of liver metastases among the different groups (*n* = 5). (G) Representative liver images and H&E staining images of CRC metastasis in liver tissue (*n* = 5). Scale bar: 1 cm, 2 mm. (H, I) Representative images of IF staining for MPO (red), Cit‐H3 (green), and DAPI (blue) in liver tumor tissue from a murine splenic liver metastasis model (H) and quantification of NETs among different groups (*n* = 5) (I). Scale bar, 50 µm. All the data are shown as the mean ± SD; ^*^, *p* < 0.05; ^**^, *p* < 0.01; ^***^, *p* < 0.001. NS, not significant. Statistical significance was assessed using two‐way ANOVA.

## 3 Discussion

In the current study, the high density of TANs and elevated NETs levels in primary CRC were correlated with cancer progression and poor prognosis, which is consistent with the findings of previous studies [27, 45]. Nevertheless, the functional plasticity of TANs allows them to exert both antitumorigenic (N1 phenotype) and protumorigenic (N2 phenotype) effects [46], and the expression of *CCL2, MMP9*, and *Arg1* is typically used to indicate the N2 phenotype, whereas the expression of *Fas*, *ICAM1*, and *TNF‐α* is associated with the N1 phenotype [47]. Our RNA‐seq data suggest that CM from MIIP‐knockdown CRC cells promoted neutrophil polarization toward the N2 phenotype and NETs formation, which further promoted the migration and invasion of CRC cells. These findings indicate that the N2 phenotype of TANs might be increased in the CRC TME and that MIIP downregulation could promote CRC progression partially through the induction of TANs infiltration and N2 polarization as well as NETs formation.

Neutrophil recruitment and NETs formation are regulated by multiple factors, including cytokines, immune cell interactions, and metabolic reprogramming [44, 48]. In the present study, we identified IL‐8 as a key cytokine in MIIP‐regulated TANs infiltration and NETs formation and demonstrated that the effects of MIIP were reversed by blocking IL‐8. Neutrophils are chronically activated and generate pathogenic NETs through the IL‐8‐CXCR2 pathway in biliary atresia [49]. Lymphatic endothelial cells secrete CXCL8 and CXCL2 in response to extracellular vesicles, inducing neutrophil accumulation and NETs formation to facilitate lymph node metastasis [50]. In colon cancer, *SKAP1* was reported to increase the expression of *CXCL8* via *NFATc1*, which promoted neutrophil infiltration, NETs formation, and tumor growth [34]. Taken together, our findings indicate that IL‐8 is a crucial inducer of TANs infiltration and NETs formation in the CRC tumor microenvironment.

The expression of IL‐8 is regulated both transcriptionally and via epigenetic modifications, with the former being the primary mechanism [51]. The tumor suppressor *CSMD1* acts as an inhibitor of TNF‐induced NF‐κB and STAT3 signaling, thereby suppressing IL‐6/IL‐8 secretion and attenuating neuroinflammation in glioma [52]. TRAIL death receptors (DR4/DR5) promote constitutive and inducible IL‐8 secretion through the NF‐κB p65 and ERK/MAPK pathways, contributing to non‐small cell lung cancer aggressiveness [53]. In the present study, transcriptome analysis revealed enrichment of the NF‐κB pathway in CRC cells with low MIIP expression. MIIP knockdown enhanced NF‐κB p65 phosphorylation, IL‐8 secretion, neutrophil migration, and NETs formation, and all these effects were reversed by the addition of an NF‐κB inhibitor. Collectively, these findings establish that MIIP negatively regulates the NF‐κB–IL8–TANs/NETs axis in colorectal cancer.

The activation of the NF‐κB signaling pathway can be regulated by multiple signals [54]. Chen et al. demonstrated that PKCε‐mediated phosphorylation of MIIP at Ser303 (tumor‐promoting action) induced its nuclear localization and promoted NF‐κB activation by preventing HDAC6‐dependent deacetylation of RelA/p65, focusing on the effect of MIIP on CRC cells themselves [10]. In this study, we identified PKR as a novel interacting partner of MIIP through IP‐MS screening and Co‐IP. We further revealed that a region of MIIP (amino acid 313–388) binds to the N‐terminal (amino acid 1–265) region of PKR, which has been previously characterized as the IKKβ‐binding domain [55]. Further assays revealed that MIIP suppressed PKR expression and reduced the PKR–IKKβ interaction, thereby blocking IKK activation and subsequent downstream NF‐κB signaling. Traditionally, PKR has been recognized as a central mediator of antiviral immunity and cellular stress responses [56, 57]. However, our findings broaden its functional repertoire in cancer biology: MIIP was identified as an interactor of PKR, negatively regulating the NF‐κB/IL‐8 axis that promotes neutrophil recruitment and NETs formation in CRC. Our study and that of Chen et al. delineate the functionally opposing roles of MIIP in NF‐κB regulation: phosphorylated MIIP acts as a pro‐metastatic transcriptional co‐activator, whereas MIIP serves as an antitumor signaling suppressor that constrains tumor–microenvironment crosstalk.

Recently, NETs degradation has represented a potent strategy for suppressing cancer progression and metastasis [43]. DNase I (Pulmozyme) has been approved by the US Food and Drug Administration for the treatment of cystic fibrosis [31]. DNase I treatment significantly decreased pulmonary metastasis in mouse models of breast cancer [30]. Additionally, in mouse hepatocellular carcinoma and CRC models, DNase I increased the efficacy of anti‐PD‐1 therapy by inhibiting NETs [58, 59]. In this study, we demonstrated that DNase I treatment significantly suppressed CRC tumor cell metastasis in vivo with a favorable safety profile, especially in CRC with relatively high MIIP expression. These findings indicate that the use of DNase I might be a potential therapeutic strategy in CRC and that the expression of MIIP may serve as a predictive biomarker for patient stratification, particularly in the context of DNase I‐based interventions targeting NET‐dependent metastasis in colorectal cancer.

However, some limitations of this study should be acknowledged. First, our analysis of TANs was limited to the conventional N1–N2 dichotomy, which precluded a more refined exploration of their phenotypic complexity. Second, we did not clarify the precise molecular mechanisms underlying NET‐induced CRC progression. Moreover, we investigated only the therapeutic potential of DNase I in animal models with small sample sizes, and the exact mechanism underlying this phenomenon needs to be explored. These unresolved questions necessitate in‐depth investigation and future preclinical and clinical studies.

## 4 Conclusion

In summary, the current findings revealed the negative regulatory effect of MIIP in CRC cells on neutrophil infiltration and NETs formation in the CRC TIME. Mechanistically, we first propose that MIIP in CRC cells decreases TANs infiltration and NETs formation via suppressing PKR‐mediated NF‐κB p65/IL‐8 signaling activation, thereby inhibiting tumor cell metastasis. In addition, the present study suggests that the use of DNase I might be a promising therapeutic strategy for CRC and that MIIP is a potential marker for patient stratification (Figure 7).

**FIGURE 7:**
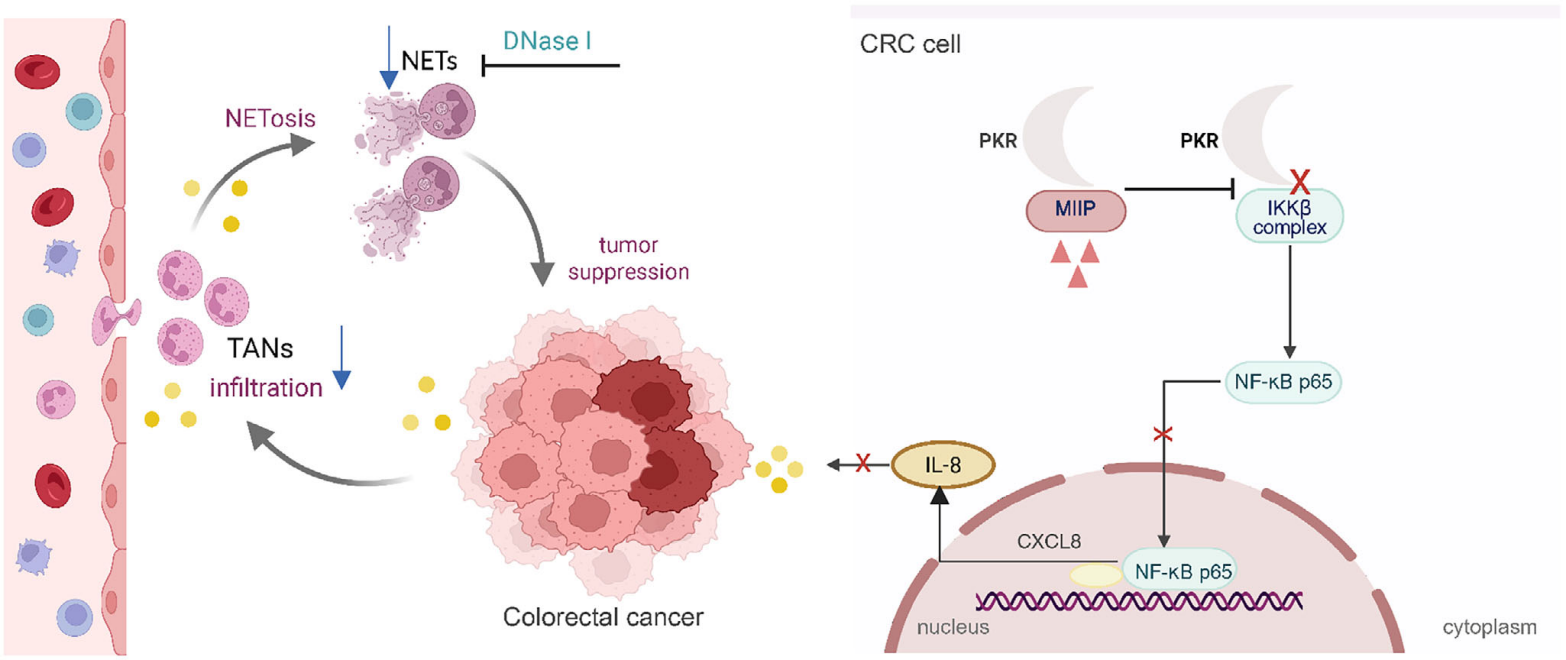
Schematic illustration shows that MIIP inhibits colorectal cancer progression by decreasing TANs infiltration and NETs formation through the PKR/NF‐κB p65/IL‐8 axis, providing potential therapeutic strategies for colorectal cancer.

## 5 Experimental Section

### 5.1 Clinical Samples

We retrospectively collected 400 formalin‐fixed and paraffin‐embedded (FFPE) CRC specimens from patients who underwent radical CRC surgery at Tianjin Medical University Cancer Institute and Hospital from July 2014 to October 2018 (Tianjin cohort). All the cases were pathologically confirmed. All clinical specimens were obtained with the patients’ consent, and the study was approved by the Ethics Committee of Tianjin Medical University Cancer Hospital (Approval No. EK201811). Our research adhered to the principles outlined in the Declaration of Helsinki.

### 5.2 Bioinformatics Analyses of Public Databases

TCGA‐COAD and TCGA‐READ gene expression and clinical data were downloaded from TCGA, with RNA‐seq data extracted in FPKM format (https://portal.gdc.cancer.gov). Immune infiltration was estimated using CIBERSORT (LM22 signature matrix) via CIBERSORT.R script (https://cibersortx.stanford.edu/). The correlations between MIIP expression and immune cell proportions were analyzed and visualized as plots using ggplot2 (v3.4.4) in R (v4.2.1). The protein structures of MIIP(AF‐Q5JXC2‐F1) and PKR (AF‐P19525‐F1), obtained from the AlphaFold site (https://alphafold.ebi.ac.uk/), were subjected to molecular docking using HDOCK. The resulting complex was subsequently visualized with PyMOL and LigPlot for structural and interaction analysis.

### 5.3 Stable Cell Line Establishment

Cell lines were purchased from the Cell Bank of Type Culture Collection of the Chinese Academy of Sciences (Shanghai, China). SW620 (RRID: CVCL_0547) and SW480 (RRID: CVCL_0546) cells were maintained in L‐15 medium, HCT116 (RRID: CVCL_0291) cells were maintained in McCoy's 5A medium, HEK‐293T (RRID: CVCL_0063) cells were maintained in DMEM, and CT‐26 (RRID: CVCL_7254) and MC‐38 (RRID: CVCL_B288) cells were maintained in RPMI 1640 medium supplemented with 10% FBS. Lentiviral vectors encoding human MIIP or short hairpin RNAs (shMIIP‐1: 5'‐GTGGAGGAAGACCATGAATGC‐3'; shMIIP‐2: 5'‐GGTGAGGGATTCTGTGGAAGT‐3'); murine MIIP or shMIIP (shMIIP‐1:5'‐CAAAGAGGATTGTGTTTGCAA‐3'; shMIIP‐2: 5'‐CTTCTCTATGCTGCAGAGGTT‐3'); and human PKR or short hairpin RNAs (shPKR‐1: 5'‐GCTGAACTTCTTCATGTATGT‐3'; shPKR‐2: 5'‐GAGGCGAGAAACTAGACAAAG‐3') were purchased from Genechem (Shanghai, China), along with their corresponding control viruses. CRC cells were infected with different lentiviruses and selected in medium supplemented with puromycin or blasticidin following the manufacturer's protocol. All the cell lines were cultured in a humidified atmosphere containing 5% CO_2_ at 37°C. All the cell lines used in this study were tested negative for mycoplasma contamination.

### 5.4 Human Neutrophil Isolation

Neutrophils were isolated from the peripheral blood of healthy volunteers using density gradient centrifugation with a neutrophil isolation solution (1895; Serumwerk Bernburg AG, Germany). Neutrophils were cultured in RPMI 1640 medium supplemented with 10% FBS.

### 5.5 Neutrophil Chemotaxis Assay

The neutrophil chemotaxis assay was performed using a transwell coculture system (3415; Corning Costar, USA). Neutrophils (2 × 10^5^ cells) were added to the upper chamber, and CM from CRC cells was added to the lower chamber. In the rescue assay, neutralizing antibodies against IL‐8 (10 mg/mL, MAB208, R&D, USA; RRID: AB_2249110) or IL‐8 protein (10 ng/mL, HY‐P7224, MCE, USA) were added to the CRC CM in the lower chamber for the rescue assay. CRC cells were treated with an NF‐κB inhibitor (10 µM, Bay 11–7082, MCE, USA). After 2 h of incubation, the neutrophils that migrated into the lower chamber were counted [30].

### 5.6 Neutrophil Apoptosis Assay

Neutrophils were stained with an Annexin V‐APC/7‐AAD apoptosis kit (AP105; MULTI SCIENCE, China) according to the manufacturer's instructions after they were co‐cultured with CRC CM at 37°C for 24 h. The apoptosis rate was determined using a BD FACSCanto II flow cytometer (BD Biosciences, USA).

### 5.7 RNA‐Seq

CRC cells (*n* = 3 per group) and CRC CM‐treated neutrophils (*n* = 3 per group) were collected for RNA‐seq, which was performed by Novogene Corporation (Beijing, China). Briefly, messenger RNA was purified from total RNA using poly‐T oligo‐attached magnetic beads. Libraries were constructed from the purified mRNA and sequenced on the Illumina HiSeq platform, generating 150‐base‐pair paired‐end reads. Using HTSeq v0.6.0, read counts and fragments per kilobase million (FPKM) mapped reads for each sample were generated. FPKM was used to screen genes that were differentially expressed between pairwise comparisons. DEGs were identified using |Log_2_ fold change| > 1 and adjusted p‐value (FDR) < 0.05 as the threshold.

### 5.8 ELISA

MPO‐DNA complexes were evaluated using previously described ELISA methods [30]. Briefly, 96‐well ELISA plates were coated with 5 mg/mL anti‐MPO (22225‐1‐AP; Proteintech, China; RRID: AB_2879037) antibody at 4°C for 16 h and blocked with 1% BSA at room temperature for 45 min. Then, 50 µL of CRC CM mixed with peroxidase‐labeled anti‐DNA monoclonal antibody (11774425001; Roche, USA; RRID: AB_2909412) was added and incubated for 2 h at room temperature. Afterward, peroxidase substrate (11774425001; Roche, USA) was added, and the samples were incubated in the dark at 4°C for 45 min. The absorbance at 405 nm was measured with a microplate reader. IL‐8 in the CM was quantified using a Human IL‐8/CXCL8 ELISA Kit (abs510004; Absin, China) or a Mouse CXCL1/KC (IL‐8) ELISA Kit (abs520017; Absin, China) following the manufacturer's guidelines.

### 5.9 Cytokine Array

Cytokine detection in CRC CM was performed using a Proteome Profiler Human Cytokine Array Kit (ARY005B; R&D Systems, USA) according to the manufacturer's protocol.

### 5.10 CRC Cell Migration and Invasion Assays

CRC cells (2 × 10^5^) were seeded into the upper chamber (3422; Corning, USA) with or without Matrigel coating (1:8; 354248; Corning, USA). NETs (induced by PMA, 20 nM; P1585; Sigma) were added to the lower chamber with or without DNase I (1 mg/mL; D4527; Sigma, USA) treatment. After 48 h, the CRC cells remaining in the upper chamber were carefully removed. Afterward, the upper chamber was fixed with 4% paraformaldehyde and stained with 0.2% crystal violet. The number of invaded or migrated cells was counted in 5 random fields (200×).

### 5.11 Cell Proliferation Assay

Cell proliferation was assessed using a Cell Counting Kit‐8 (CCK‐8) kit (GK10001; GLP Bioscience, USA). CRC cells were seeded in 96‐well plates, incubated for 8 h, and then cultured with the corresponding CM. CCK‐8 solution was added, and the absorbance was measured daily at 450 nm using a microplate reader.

### 5.12 Western Blotting

Protein was extracted from CRC cells, separated by SDS–PAGE, and transferred to a PVDF membrane. The membrane was then blocked with 5% skim milk for 1 h and incubated with primary antibody overnight at 4°C. The primary antibodies used were as follows: anti‐β‐Actin (1:1000, 4967, CST, USA; RRID: AB_330288), anti‐LaminB1 (1:1000, 12586, CST, USA; RRID: AB_2650517), anti‐MIIP (1:1000, HPA044948, Sigma, USA; RRID: AB_10795887), anti‐MIIP (1:1000, orb537083, Biobyrt, USA; RRID: AB_3665851), anti‐PKR (1:200, SC‐6282, Santa Cruz; RRID: AB_628150), anti‐NF‐κB p65 (1:1000, 8242, CST, USA; RRID: AB_10859369), and anti‐phospho‐NF‐κB p65 (Ser276) (1:1000, AF3387, Affinity, China). Afterward, the membranes were incubated with anti‐rabbit/mouse IgG secondary antibodies (1:5000, 7074 or 7076; CST, USA; RRID: AB_2099233 or RRID: AB_330924) at room temperature for 1 h. Enhanced chemiluminescence reagent (E‐IR‐R307; Elabscience, China) was used for signal detection after secondary antibody incubation.

### 5.13 RNA Extraction and RT–qPCR

Total RNA was isolated from the cells using TRIzol (15596026; Invitrogen, USA) and chloroform (CX1060; Merck, Germany). cDNA was synthesized using a PrimeScript RT reagent kit (RR037A; Takara, Japan). RT–qPCR was performed with a TB Green Premix Ex Taq GC Kit (RR071B; Takara, Japan) and a StepOne Plus Real‐Time PCR System (4376600; Thermo, USA). The primer sequences are listed in Table S3. Relative gene expression was calculated using the 2^−ΔΔCt^ method and normalized to that of GAPDH.

### 5.14 Co‐IP

Co‐IP assays were conducted using a Pierce Classic Magnetic IP/Co‐IP Kit (88804; Thermo Fisher Scientific, USA) following the manufacturer's instructions. The antibodies used were as follows: anti‐PKR (SC‐6282; Santa Cruz; RRID: AB_628150), anti‐DYKDDDDK tag (14793; CST, USA; RRID: AB_2572291), anti‐Flag‐Tag (T519; Signalway Antibody, USA), anti‐HA‐Tag (3724; CST, USA; RRID: AB_1549585), anti‐normal rabbit IgG (2729; CST, USA; RRID:AB_1031062), and anti‐mouse IgG (5873; CST, USA; RRID: AB_10835692).

### 5.15 IP–MS

Flag‐tagged proteins were captured using ANTI‐FLAG M2 Magnetic Beads (M8823; Sigma, USA; RRID: AB_2637089), and immunoprecipitation was performed using a Pierce Classic Magnetic IP/Co‐IP Kit (88804; Thermo Fisher Scientific, USA). The separated immunoprecipitates were analyzed via Orbitrap Eclipse mass spectrometry at the Liquid Chromatography and Mass Spectrometry Analysis Platform, Tianjin Medical University.

### 5.16 Animal Model

Six‐week‐old female BALB/c mice were purchased from Vital River Laboratory Animal Technology Company (Beijing, China). For the orthotopic CRC mouse model, the mice were randomly divided into two groups (the CT26‐vector group and the CT26‐MIIP group; *n* = 5 in each group). As described previously [11], under inhalation anesthesia with isoflurane, the mice were injected with 1 × 10^6^ CT26 cells in 50 µL of PBS into the cecum wall. The mice were monitored weekly and sacrificed after 28 days. For the CRC liver metastasis mouse model, stably transfected luciferase‐tagged CT26 cells with different levels of MIIP expressions (vector or MIIP‐OE) were used for intrasplenic injections as described in a previous study [19]. The mice were randomly divided into four groups (*n* = 5 in each group) on the basis of MIIP expression (vector or MIIP‐OE) and DNase I treatment. For DNase I treatment, mice were intraperitoneally injected with DNase I (5 mg/kg; Sigma) 2 h before CRC cell injection, followed by daily injection of DNase I for 9 days and then maintenance injections twice per week [30]. Tumor BLI was monitored weekly using an IVIS Lumina III Imaging System (PerkinElmer, USA) for 21 days prior to sacrifice. All animal procedures were approved by the Animal Ethics Committee of Tianjin Medical University (Approval No: TMUaMEC2024011).

### 5.17 Immunohistochemical (IHC) Staining

Briefly, 4‐µm sections from FFPE tissue were incubated with antibodies overnight at 4°C after routine deparaffinization, rehydration, and antigen retrieval in EDTA (pH = 8.0). The antibodies used included anti‐CD66b (1:200, 305102, Biolegend, USA; RRID: AB_314494), anti‐MIIP (1:200, HPA044948, Sigma, USA; 1:200, 20630‐AP, Proteintech, China; RRID: AB_10795887), anti‐Ly6G (1:500, 127601, Biolegend, USA; RRID: AB_1089179), anti‐CXCL1 (1:100, 12335‐1‐AP, Proteintech, China), and phospho‐NF‐κB p65 (Ser276) (1:200, AF3387, Affinity, China). The sections were then incubated with a secondary antibody tagged with peroxidase enzyme (PV9000; ZSGB‐BIO, China) for 30 min and visualized with 0.05% DAB. The tumor cells were identified as positive for MIIP and IL‐1/IL‐8 when yellow–brown staining was localized in the cytoplasm, and as positive for phospho‐NF‐κB p65 when yellow–brown staining was localized in the nucleus. Scoring was performed under a microscope on the basis of the staining intensity (0–3) and positive cell proportion (0–3), and multiplied to yield a total score (0–9). Score ranges: 0 = negative, 1–3 = weak, 4–6 = moderate, 9 = strong. Specifically, MIIP scores 0–4 were considered low‐expression, and those of 6–9 were considered high‐expression. CD66b positivity was defined as yellow–brown staining on the neutrophil cell membrane. The density of neutrophils in CRC tissue was quantified by counting the number of CD66b‐stained cells in 5 randomly selected high‐power fields (200×). The median cutoff was used to classify CD66b‐positive neutrophil infiltration into high and low groups.

### 5.18 Immunofluorescence (IF)

For IF staining of tumor tissue, after deparaffinization, rehydration, and antigen retrieval, 4 µm FFPE tissue sections were incubated with primary antibodies overnight at 4°C. The primary antibodies anti‐Cit‐H3 (1:200; AM10179; Origen, USA; RRID: AB_11214865), anti‐MPO (1:500; AF3667; R&D, USA; RRID: AB_2250866), and anti‐MIIP (1:200; HPA044948; Sigma, USA; RRID: AB_10795887) were used according to the respective instructions. The sections were then incubated with anti‐mouse IgG‐Alexa Fluor 488 (1:200, abs20013, Absin, China), donkey anti‐rabbit IgG‐Alexa Fluor 594 (1:200, abs20021, Absin, China) or donkey anti‐goat IgG‐Alexa Fluor 647 (1:200, abs2002, Absin, China) for 1 h at room temperature in the dark. The sections were stained with DAPI and visualized by confocal laser microscopy (ZEISS LSM 880, Germany; RRID: SCR_020925). The median cutoff was used to classify the NETs area into high and low groups.

For the detection of NETs by double‐immunofluorescence staining for Cit‐H3 and MPO, 2.5 × 10^5^ neutrophils were seeded on a coverslip and cultured with CRC CM in 24‐well plates at 37°C for 12 h. The cells were fixed with 4% paraformaldehyde for 15 min and permeabilized with 0.1% Triton X‐100 for 10 min. The cells were then blocked with 5% BSA and incubated with anti‐Cit‐H3 (1:200; AM10179; Origen, USA; RRID: AB_11214865) and anti‐MPO (1:500; AF3667; R&D, USA; RRID: AB_2250866) at 4°C overnight. The cells were incubated with fluorochrome‐conjugated secondary antibodies for 1 h and counterstained with DAPI (abs9235; Absin, China) in the dark at room temperature. Afterward, the slides were observed with a confocal laser microscope.

For the detection of dsDNA of NETs formation, the coverslip with neutrophils cultured with CRC CM was fixed in 4% paraformaldehyde for 20 min at room temperature and then incubated with SYTOX Green (1 µM; S7020; Thermo Fisher Scientific, USA) for 2 h at 37°C. The nuclei were stained with DAPI in the dark at room temperature. Afterward, the slides were analyzed using a confocal laser microscope.

### 5.19 Statistical Analyses

Data analyses were performed using GraphPad Prism 7.0 (GraphPad Software, Inc., USA) and SPSS 27.0 (IBM Corp., USA). CRC patients’ survival data were analyzed with Kaplan–Meier plots and log‐rank tests. Pearson's χ^2^ test and Fisher's exact test were used to assess the associations between MIIP/TANs/NETs and clinicopathological characteristics. The experimental data are expressed as the mean ± SDs. Student's t‐test or the Mann‐Whitney U test was used to evaluate the differences between two groups. Multiple groups were compared using one‐way ANOVA or two‐way ANOVA. The significance levels are denoted as ^*^, *p* < 0.05; ^**^, *p* < 0.01. ^***^, *p* < 0.001. NS, not significant.

## Author Contributions

J.L. and S.L. analyzed the data and wrote the manuscript. J.L. and J.D. performed the experiments with assistance from S.W., H.L., L.Z., and J.C. X.S. performed the bioinformatics analyses. Y.S. contributed to the study conception, revised the manuscript, and supervised the study. All the authors have read and approved the final manuscript.

## Conflicts of Interest

The authors declare no conflicts of interest.

## Supporting information




**Supporting File**: advs78063‐sup‐0001‐SuppMat.docx.

## Data Availability

The data that support the findings of this study are available from the corresponding author upon reasonable request.
