# Supplementary material for: MIIP Inhibits Colorectal Cancer Progression by Modulating Neutrophils Infiltration and Neutrophil Extracellular Trap Formation
Source: Adv Sci (Weinh). 2026 Sep 29:e78063. Online ahead of print. doi: 10.1002/advs.78063 (PMC13624310; doi:10.1002/advs.78063)
Supplement: Supplementary file 1 [file ADVS-9999-e78063-s001.docx]

**Figure S1.** Correlations between MIIP expression and the enrichment of neutrophils and NETs gene sets in the TCGA databases.

A) Lollipop chart of correlations between MIIP and markers of 22 immune cell types using the CIBERSORT algorithm based on TCGA CRC databases. B) The correlation between MIIP expression and the enrichment of neutrophils was analyzed using data from public TCGA CRC databases. C) The correlation between NETs-related gene signatures and MIIP expression was investigated in TCGA CRC and pancancer datasets.

**Figure S2.** Establishment of human CRC cells and detection of CRC cells and neutrophils in the coculture system.

A) Western blotting was performed to determine MIIP expression in 17 CRC cell lines. The gray intensity ratio of MIIP to β-Actin was calculated using ImageJ software. B, C) Construction and validation of human CRC cell lines with stable overexpression, knockdown, and control of MIIP. D) Human neutrophils were isolated from the peripheral blood of healthy adults, and their purity was confirmed by Giemsa staining. E) Representative flow cytometry scatter plots of the apoptosis assay result for neutrophils after coculture with CM from CRC cells with different levels of MIIP expression (n = 3). F) Representative images and statistical analysis of IF staining with SYTOX Green dsDNA dye for neutrophils after culture with CM from CRC cells with different levels of MIIP expression (n = 5). Scale bar, 50 μm. G) Principal component analysis score plot of RNA-seq data indicating discrimination between Neu-HCT116 shNC-CM and Neu-HCT116 shMIIP-CM (n = 3 per group). Volcano plot of the RNA-Seq data used to compare gene expression between Neu-HCT116 shNC-CM and Neu-HCT116 shMIIP-CM. H, I) Representative images of Transwell migration and invasion assays for CRC cells co-cultured with or without NETs. Scale bar, 500 μm. J) CCK-8 assay measuring the effect of NETs on CRC cell proliferation/viability (n = 3). All the data are shown as the mean ± SD; *, *P* < 0.05; **, *P* < 0.01; ***, *P* < 0.001. NS, not significant. Statistical significance was assessed using Student's t-test and one-way ANOVA in (C, J).

**Figure S3.** MIIP inhibits NF-κB p65-IL8 signaling in CRC cells.

A) The levels of NF-κB p65 and phosphorylated NF-κB p65 were determined by cytosolic and nuclear Western blotting B) RT–qPCR analysis of *CXCL1* (the murine homolog of human *CXCL8*) mRNA expression in cells with different levels of MIIP expression (n = 3). C) ELISA for IL-1 (the murine homolog of human IL-8) protein in CM from mouse CRC cells with different levels of MIIP expression (n = 3). D) Western blotting of key proteins involved in the NF-κB p65 signaling pathway with different levels of MIIP expression in mouse CRC cells. All the data are shown as the mean ± SD; ***, *P* < 0.001. Statistical significance was assessed using Student's t-test and one-way ANOVA in (B, C).

**Figure S4.** MIIP interacts with PKR to regulate NETs formation.

A) Schematic representation of the PKR peptide structure determined by IP–MS. B) The binding site of MIIP and PKR was predicted using HDOCK molecular docking software. C) Representative images of IF staining for MPO (red), CitH3 (green) and DAPI (blue) to show NETs formation. Scale bar, 50 μm.

**Figure S5.** A, B) Representative images of Transwell assays of CRC cells with varying levels of MIIP expression following NETs coculture and DNase I treatment. Scale bar, 500 μm. C) Changes in body weight in each group of mice after treatment (n = 5 per group, Day 28). D) H&E staining of the brain, heart, kidney and lung tissue of BALB/C mice in each group. Scale bar, 100 μm. Statistical significance was assessed using two-way ANOVA.

**Table S1.** The correlations between MIIP expression, CD66b^+^TANs and clinicopathological parameters in 400 CRC patients

| **Clinicopathological  parameter** | **Total (n=400)** | **MIIP low  expression (n=299)** | **MIIP high  expression (n=101)** | **χ²** | ***P*** | **CD66b+TANs  low density  (n=277)** | **CD66b+TANs high density   (n=123)** | **χ²** | ***P*** |
| --- | --- | --- | --- | --- | --- | --- | --- | --- | --- |
| **Gender** |  |  |  | 1.069 | 0.301 |  |  | 0.003 | 0.953 |
| Male | 204 (51.0) | 148 (49.5) | 56 (55.4) |  |  | 141 (50.9) | 63 (51.2) |  |  |
| Female | 196 (49.0) | 151 (50.5) | 45 (44.6) |  |  | 136 (49.1) | 60 (48.8) |  |  |
| **Age (years)** |  |  |  | 0.003 | 0.955 |  |  | 0.067 | 0.796 |
| ≤56 | 201 (50.2) | 150 (50.2) | 51 (50.5) |  |  | 138 (49.8) | 63 (51.2) |  |  |
| ＞56 | 199 (49.8) | 149 (49.8) | 50 (49.5) |  |  | 139 (50.2) | 60 (48.8) |  |  |
| **Tumor site** |  |  |  | 1.403 | 0.499 |  |  | 1.314 | 0.519 |
| Left hemicolon | 115 (28.7) | 83 (27.8) | 32 (31.7) |  |  | 75 (27.1) | 40 (32.5) |  |  |
| Right hemicolon | 233 (58.3) | 174 (58.2) | 59 (58.4) |  |  | 166 (59.9) | 67 (54.5) |  |  |
| Rectum | 52 (13.0) | 42 (14.0) | 10 (9.9) |  |  | 36 (13.0) | 16 (13.0) |  |  |
| **Depth of invasion** |  |  |  | 7.043 | 0.008 |  |  | 0.395 | 0.530 |
| T1~T2 | 253 (63.2) | 178 (59.5) | 75 (74.3) |  |  | 178 (64.3) | 75 (61.0) |  |  |
| T3~T4 | 147 (36.8) | 121 (40.5) | 26 (25.7) |  |  | 99 (35.7) | 48 (39.0) |  |  |
| **Lymph node metastasis** |  |  |  | 2.624 | 0.105 |  |  | 2.472 | 0.116 |
| N0 | 64 (16.0) | 53 (17.7) | 11 (10.9) |  |  | 39 (14.1) | 25 (20.3) |  |  |
| N1~N2 | 336 (84.0) | 246 (82.3) | 90 (89.1) |  |  | 238 (85.9) | 98 (79.7) |  |  |
| **Distant metastasis** |  |  |  |  |  |  |  |  |  |
| M0 | 374 (93.5) | 274 (91.6) | 100 (99.0) | 6.75 | 0.009 | 264 (95.3) | 110 (89.4) | 4.839 | 0.028 |
| M1 | 26 (6.5) | 25 (8.4) | 1 (1.0) |  |  | 13 (4.7) | 13 (10.6) |  |  |
| **TNM stage** |  |  |  |  |  |  |  | 1.829 | 0.176 |
| I~II | 241 (60.2) | 166 (55.5) | 75 (74.3) | 11.07 | ＜0.001 | 173 (62.5) | 68 (55.3) |  |  |
| III~IV | 159 (39.8) | 133 (44.5) | 26 (25.7) |  |  | 104 (37.5) | 55 (44.7) |  |  |

**Table S2.** The correlation between NETs and clinicopathological parameters in 50 CRC patients

| **Clinicopathological  parameters** | **Total (n=50)** | **NETs-low  (n=23)** | **NETs-high  (n=27)** | **χ²** | ***P*** |
| --- | --- | --- | --- | --- | --- |
| **Gender** |  |  |  | 0.725 | 0.395 |
| Male | 25 (50.0) | 13 (56.5) | 12 (44.4) |  |  |
| Female | 25 (50.0) | 10 (43.5) | 15 (55.6) |  |  |
| **Age (years)** |  |  |  | 0.593 | 0.441 |
| ≤56 | 21 (42.0) | 11 (47.8) | 10 (37.0) |  |  |
| ＞56 | 29 (58.0) | 12 (52.2) | 17 (63.0) |  |  |
| **Tumor site** |  |  |  | 0.613 | 0.736 |
| Left hemicolon | 15 (30.0) | 8 (34.8) | 7 (25.9) |  |  |
| Right hemicolon | 29 (58.0) | 12 (52.2) | 17 (63.0) |  |  |
| Rectum | 6 (12.0) | 3 (13.0) | 3 (11.1) |  |  |
| **Depth of invasion** |  |  |  | 2.853 | 0.091 |
| T1~T2 | 33 (66.0) | 18 (78.3) | 15 (55.6) |  |  |
| T3~T4 | 17 (34.0) | 5 (21.7) | 12 (44.4) |  |  |
| **Lymph node metastasis** |  |  |  | 0.227 | 0.599 |
| N0 | 8 (16.0) | 3 (13.0) | 5 (18.5) |  |  |
| N1~N2 | 42 (84.0) | 20 (87.0) | 22 (81.5) |  |  |
| **Distant metastasis** |  |  |  | / | 0.014* |
| M0 | 40 (80.0) | 22 (95.7) | 18 (66.7) |  |  |
| M1 | 10 (20.0) | 1 (4.3) | 9 (33.3) |  |  |
| **TNM stage** |  |  |  | 7.177 | 0.007 |
| I~II | 29 (58.0) | 18 (78.3) | 11 (40.7) |  |  |
| III~IV | 21 (42.0) | 5 (21.7) | 16 (59.3) |  |  |
| **CD66b^+^TANs** |  |  |  | 17.846 | ＜0.001 |
| Low density | 23 (46.0) | 18 (78.3) | 5 (18.5) |  |  |
| High density | 27 (54.0) | 5 (21.7) | 22 (81.5) |  |  |

*Data from Fisher's exact test.

**Table S3.** Primer sequences for RT–qPCR

| Species | Gene | Sequence (5' to 3') |
| --- | --- | --- |
| Homo sapiens | *MIIP* | Forward: ATCTGCAGCCATCCTGAACC  Reverse: CAGTGTGTCAGAGGCGTCAA |
| Homo sapiens | *CXCL8* | Forward: ACTCCAAACCTTTCCACCC  Reverse: ACTTCTCCACAACCCTCTGC |
| Homo sapiens  Mus musculus | *GAPDH*  *mMIIP* | Forward: TTGATTTTGGAGGGATCTCG  Reverse: GGGGACTTCACGTTCACACT  Forward: CACTAGTGAGCCCGAGGCCT  Reverse: CCTTATAGTCCTTATCATCGTC |
| Mus musculus  Mus musculus | *mCXCL1*  *mGAPDH* | Forward: CCGCTCGCTTCTCTGTGCAG  Reverse: GGGCAACACCTTCAAGCTCTGG  Forward: AGGTCGGTGTGAACGGATTTG  Reverse: GGGGTCGTTGATGGCAACA |
